# Molecular docking and machine learning analysis of Abemaciclib in colon cancer

**DOI:** 10.1186/s12860-020-00295-w

**Published:** 2020-07-08

**Authors:** Jose Liñares-Blanco, Cristian R. Munteanu, Alejandro Pazos, Carlos Fernandez-Lozano

**Affiliations:** 1 Department of Computer Science and Information Technologies, Faculty of Computer Science, University of A Coruña, CITIC, Campus Elviña s/n, A Coruña, 15071 Spain; 2 Grupo de Redes de Neuronas Artificiales y Sistemas Adaptativos. Imagen Médica y Diagnóstico Radiológico (RNASA-IMEDIR). Instituto de Investigación Biomédica de A Coruña (INIBIC). Complexo Hospitalario Universitario de A Coruña (CHUAC), Sergas. Universidade da Coruña (UDC), Xubias de arriba, 84, A Coruña, 15006 Spain

**Keywords:** Machine learning, Molecular docking, Colon cancer, Prognosis, Drug repurposing, FABP6, Abemaciclib, TCGA

## Abstract

**Background:**

The main challenge in cancer research is the identification of different omic variables that present a prognostic value and personalised diagnosis for each tumour. The fact that the diagnosis is personalised opens the doors to the design and discovery of new specific treatments for each patient. In this context, this work offers new ways to reuse existing databases and work to create added value in research. Three published signatures with significante prognostic value in Colon Adenocarcinoma (COAD) were indentified. These signatures were combined in a new meta-signature and validated with main Machine Learning (ML) and conventional statistical techniques. In addition, a drug repurposing experiment was carried out through Molecular Docking (MD) methodology in order to identify new potential treatments in COAD.

**Results:**

The prognostic potential of the signature was validated by means of ML algorithms and differential gene expression analysis. The results obtained supported the possibility that this meta-signature could harbor genes of interest for the prognosis and treatment of COAD. We studied drug repurposing following a molecular docking (MD) analysis, where the different protein data bank (PDB) structures of the genes of the meta-signature (in total 155) were confronted with 81 anti-cancer drugs approved by the FDA. We observed four interactions of interest: GLTP - Nilotinib, PTPRN - Venetoclax, VEGFA - Venetoclax and FABP6 - Abemaciclib. The FABP6 gene and its role within different metabolic pathways were studied in tumour and normal tissue and we observed the capability of the FABP6 gene to be a therapeutic target. Our in silico results showed a significant specificity of the union of the protein products of the FABP6 gene as well as the known action of Abemaciclib as an inhibitor of the CDK4/6 protein and therefore, of the cell cycle.

**Conclusions:**

The results of our ML and differential expression experiments have first shown the FABP6 gene as a possible new cancer biomarker due to its specificity in colonic tumour tissue and no expression in healthy adjacent tissue. Next, the MD analysis showed that the drug Abemaciclib characteristic affinity for the different protein structures of the FABP6 gene. Therefore, in silico experiments have shown a new opportunity that should be validated experimentally, thus helping to reduce the cost and speed of drug screening. For these reasons, we propose the validation of the drug Abemaciclib for the treatment of colon cancer.

## Background

Colon adenocarcinomas (COAD) significantly contributes to mortality and morbidity [1] of cancer in the world population. In 2018, of the approximately 18 million new cases, about 10% were colorectal cancer (1.8 million cases), according to data from the World Cancer Research Fund. This type of cancer gains significance when we focus on data within Spain, as it is the primary cause of hospital stay in the country. It is estimated that by 2019 there will be around 44,000 new cases of COAD. Moreover, this problem is also alarming in Galicia, which has the fifth highest number of COAD cases, with 2,500 new cases each year according to data from the Spanish Cancer Association [2]. All studies indicate that early detection and targeted treatment are the best weapons to reduce these devastating statistics.

To achieve this goal, the scientific mass is, on the one hand, generating different types of omics data to define diseases molecularly and, on the other hand, designing different data analysis models to extract valuable information from these data.

In such context, extensive scientific contributions have based their research on data reported by international initiatives such as The Cancer Genome Atlas (TCGA) [3]. The TCGA was born with the objective of obtaining a multidimensional genomic map of the main genomic changes in a wide variety of tumours. Analysis of the data hosted by the TCGA offers scientists new opportunities to obtain highly reproducible results that can be extrapolated to most of the world’s populations. With a sample size of over 11,000 patients categorised into 33 different tumour types, this repository offers the possibility of creating models sufficiently robust for the extraction of statistically reliable results and conclusions.

With access to such an amount of data, an ideal environment is created for the use of new computational methods capable of extracting information from the data and simulating complex biological processes. Computational methods such as machine learning (ML) and molecular docking (MD) are examples that can provide new and different visions in the fight against complex diseases, such as COAD. Both techniques have already been used extensively in recent years to bring about new results and conclusions [4–9].

As far as therapeutic targets are concerned, an immense investment is being made in terms of time, personnel, resources and money, to experimentally validate new biological targets and new drugs that act efficiently on them. Once the drug to be validated has been identified, experiments are carried out to test and validate whether the drug has the expected effect on cellular and animal models. Subsequently, a clinical trial is necessary, where a significant number of patients must be recruited, all adverse aspects must be analysed and all quality controls must be passed. It is here, in the clinical trials phase, that budgets skyrocket. Therefore, it is necessary to pre-screen interactions, in a in silico way, to obtain potential candidates that can be tested later in the laboratory. This prior in silico step greatly helps to reduce experimental costs. It should be noted that one out of every 5,000 drugs goes to the clinic [10], which implies a disproportionate investment on the part of the pharmaceutical companies. Therefore, finding pathways and shortcuts from basic research to clinical research through translational research would offer a significant advantage to this field of research.

With this in mind, the present work has used public data from TCGA and has employed the latest ML and MD techniques to predict new biomarkers in COAD and simulate the effect of drugs already approved for repurposing to this type of tumour.

For some years now, promising results have been reported on the effect of several drugs on diseases for which they were not designed. For example, the drug Zidovudine, which was originally designed for the treatment of cancer, has subsequently been used for HIV/AIDS. Another example is Rituximab, which had originally been indicated for various types of cancers and has subsequently been approved for rheumatoid arthritis, or Raloxifene, which went from being used for osteoporosis to being used for breast cancer [11]. Experimentally testing all the drugs used today in all diseases is unfeasible. Fortunately, with the increase in computational capacity, techniques of drug repurposing can give a realistic approximation of what might occur in nature.

Both the results obtained by previous researchers reported in the scientific literature and the results of the in silico analysis of the present work seem to coincide that the FABP6 gene presents all the necessary characteristics to be proposed as a potential candidate for an early diagnostic marker in COAD patients. Moreover, the results of MD indicate a strong interaction between the drug Abemacicib and the different protein conformations of the FABP6 gene, leading to a possible inhibition of the protein activity.

It is known that FABP6 belongs to a group of low-molecular-weight proteins related to the transport of long-chain bioactive fatty acids in cells. In humans, there are nine different subgroups (FABP1-9). This group of proteins play a role in the development of different types of cancer cells [12–16], and have also been proposed as diagnostic markers and therapeutic targets [17–19]. Specifically, FABP6 is highly expressed in the ileum and is an intracellular transporter of bile acids in ileal epithelial cells, contributing to the catalysis and metabolism of cholesterol. In relation to COAD cells, previous works have observed that there are high concentrations of faecal bile acids, in particular, secondary bile acids [20–22]. Furthermore, the involvement of FABP6 in the development of colon cancer has been addressed in previous publications [23, 24].

This work presents two distinct phases. Firstly, a search has been carried out for previously published gene signatures obtained from TCGA data using ML algorithms. The use of these algorithms for this type of problem offers the possibility of finding patterns and identifying important variables that have not been identified with the classical techniques. Thus, after a thorough review of the different papers published under these requirements, three gene signatures with prognostic value have been identified for colon cancer [25–27]. Secondly, once our meta-signature was created, the objective was to search for and identify those genes that could behave as therapeutic targets in colon cancer and to carry out an in-depth study for their validation and contribution to new treatment approaches, in a in silico way. To this end, a repurposing of anti-cancer drugs, already approved by the FDA for use in different types of tumour s, has been carried out. The results of this work show a strong interaction, in in silico experiments, between the PDB structures of the FABP6 gene and the drug *Abemaciclib*. An in-depth study of this interaction, which is detailed in this work, offers hopeful results on a possible new treatment against colon cancer, which must be validated experimentally.

## Results

### New gene signature for COAD prognosis prediction

The first objective of this work was to search for previously published gene signatures that could predict the prognosis of COAD. In order to do this, we opted to search for these signatures based on works using Machine Learning techniques. We hypothesised that Machine Learning techniques, together with different techniques for selecting characteristics, could find variables (in this case genes) of interest that have not previously been identified by conventional techniques such as mutation analysis or differential expression analysis.

In order to avoid biases between different cohorts, such as data normalisation, as the data have been generated on different platforms, the search for the papers focused on those that used data from the TCGA repository, mainly due to its heterogeneity and its large number of samples and results published using Machine Learning techniques.

Three papers were identified [25–27] that satisfied all the requirements: they used colon cancer data from the TCGA repository, applied some type of dimensionality reduction techniques linked to Machine Learning techniques, and reported a gene signature that was capable of predicting disease prognosis with great precision.

The three signatures published by each of the selected papers are shown in Table 1.

**Table 1:** Gene signatures obtained from previous works

**Work**	**Gene signature**
Sun et al. 2018	TREML2, PADI4, NCKIPSD, PTPRN, PGLYRP1, C5orf53,
	TREML3, NOG, VIP, RIMKLB, NKAIN4, FAM171B
Xu et al. 2017	HES5, ZNF417, GLRA2, OR8D2, HOXA7, FABP6, MUSK,
	HTR6, GRIP2, KLRK1, VEGFA, AKAP12, RHEB, NCRNA00152, PMEPA1
Wen et al. 2018	GLTP, METTL7A, PPAP2A, CITED2, SCARA5, CDH3,
	IL6R, PKIB, GLP2R, LINC00974, EPB41L3, NR3C2

The three signatures were combined for later experiments, generating a meta-signature of 34 genes. None of these genes, after verification with the repository Intogen [28], was previously catalogued as a genetic driver for any type of cancer. Therefore, we considered that this set of genes could harbor some previously unidentified biomarker or therapeutic target that could predict the appearance of COAD, how the disease will develop, or inhibit its growth.

Therefore, the hypothesis of the present work is that the identification of different gene signatures, reported by different research groups, working under the same data and the same problem, may harbour genes of interest that can be used as new therapeutic targets. We will validate the signatures in different biological problems using ML techniques and conventional statistical techniques, and we will use MD techniques to identify if there is an approved drug that strongly interacts with any of the protein products of the identified genes, and coul be used for the treatment of COAD. Therefore, an experiment of repurposing drugs against these targets would be carried out to identify new therapeutic targets and their respective treatments in colon cancer.

Below are the results obtained after testing the hypothesis put forward in this section. Two experiments were carried out. Firstly, Machine Learning experiments for different classifications in order to clarify and validate the real predictive value of the signature obtained. Secondly, Molecular Docking experiments in order to search for candidates that could be possible therapeutic targets and the corresponding drugs that interact with them.

### Machine learning and statistical analysis

To validate whether the meta-signature obtained is capable of predicting different clinical outcomes, three types of Machine Learning experiments were designed: 1) classification of different stages of cancer with expression data; 2) classification of the metastatic stage in lymph nodes with expression data; 3) classification expression data of tumour and healthy adjacent samples.

These three experiments were mainly designed to observe the predictive potential of the meta-signature obtained. For the first two experiments, the obtained signature was compared with others to determine if there was sufficient information to predict advanced carcinogenic aspects. As for the third experiment, the aim was to observe whether the signature was capable of differentiating, in a significant way, between tumour and adjacent normal tissue, thus being able to identify specific omic variables in tumour tissue, and generate the possibility of finding new biomarkers or therapeutic targets specific to the tumour. The three experiments were compared with two algorithms and three different signatures. The Random Forest and Glmnet algorithms were trained for three different expression data subsets of the TCGA COAD cohort: 1) the 34 genes of the meta-signature obtained from the aforementioned studies, which will be the object of study in this work; 2) a random signature of 34 genes and 3) a signature that houses the genetic drivers for colon cancer, obtained from the Intogen repository.

#### Classification of different stages of cancer

Available data from the COAD cohort of the TCGA were downloaded. As mentioned, three different datasets were generated for each signature to study. As a dependent variable, patients were classified according to their stages. Patients were grouped into two classes (stage I-II; stage III-IV) representing the good and bad prognosis of the patients, respectively.

**Fig. 1:**
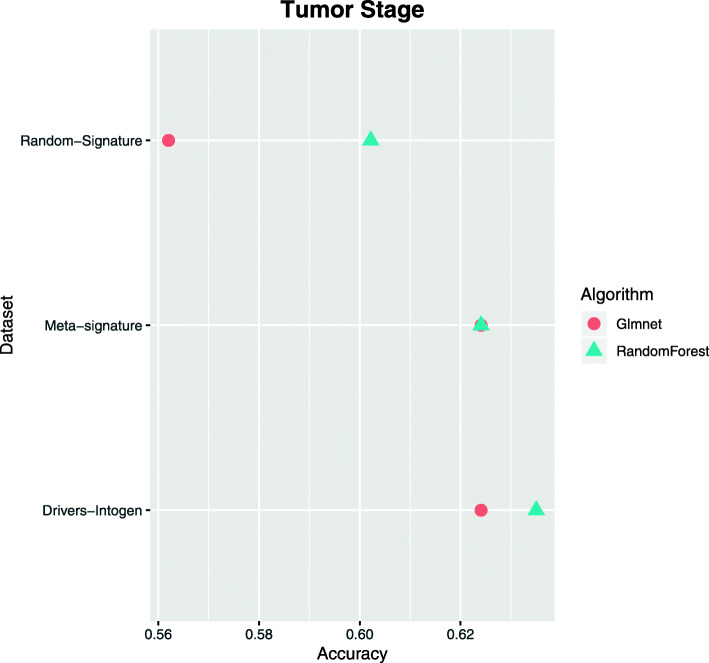
Classification according to the stage of the patients. A comparative experiment was carried out with different datasets and different algorithms for the classification of patients according to their stage. The classification consisted of a binary classification, grouping the patients in two classes (stage I-II and stage III-IV)

Figure 1 shows the results achieved by each data-algorithm binomial. The worst results were obtained with algorithms trained with random signature, as expected. As for the other two signatures, those obtained a slightly higher yield, around 2.5 points more than the best performance of the random signature. Due to the small difference in the three signatures, it can be deduced that this is an extremely complicated problem and that both the meta-signature and the genetic drivers downloaded from the Intogen repository do not present significant information about the problem to be solved. To confirm this assumption, Friedman’s statistical test was performed to see if there was any significant difference between the models. A *p*-value=0.2208 indicate that no model existed that was significantly better than the others.

#### Classification of patients according to their metastatic stage in lymph nodes

The next problem addressed was the prediction of the metastatic stage of patients. In the same way as the previous one, three different datasets were created with the same signatures of the previous problem. In this case, the patients were classified into two groups according to their metastatic stage (N0 and N1-3). This problem was established to obtain information about the very early metastasis development. To date, there is still great uncertainty about the omic variables involved in the process of metastasis, so it is also considered a very complex problem to solve.

**Fig. 2:**
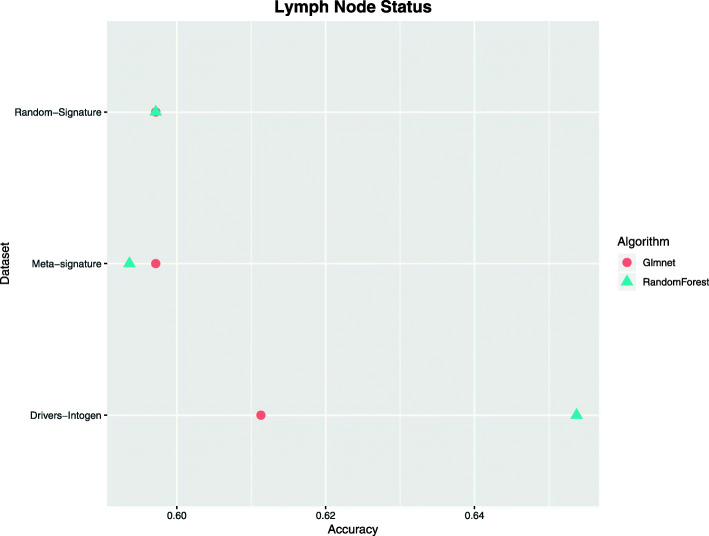
Classification by metastatic stage of patients. A comparative experiment was carried out with different datasets and different algorithms for the classification of patients according to their metastatic stage of lymphatic node. The classification consisted of a binary classification, grouping patients into two classes (stage n0 and stage 1-3)

In this case, Fig. 2 shows that the signature containing the genetic drivers is superior to the other two signatures. In this graph, we can deduce that our meta-signature does not contain any type of useful information for discerning different metastatic stages, since it has yields even lower than the random signature in the training with certain algorithms. After performing Friedman’s statistical test, a *p*-value=0.27 was obtained, indicating that no model was significantly better than the others.

#### Classification of tumour and adjacent healthy tissue

In the same way as in the previous experiments, three datasets were obtained corresponding to the three signatures used. In this case, the samples were classified between tumour samples and healthy adjacent tissue samples.

**Fig. 3:**
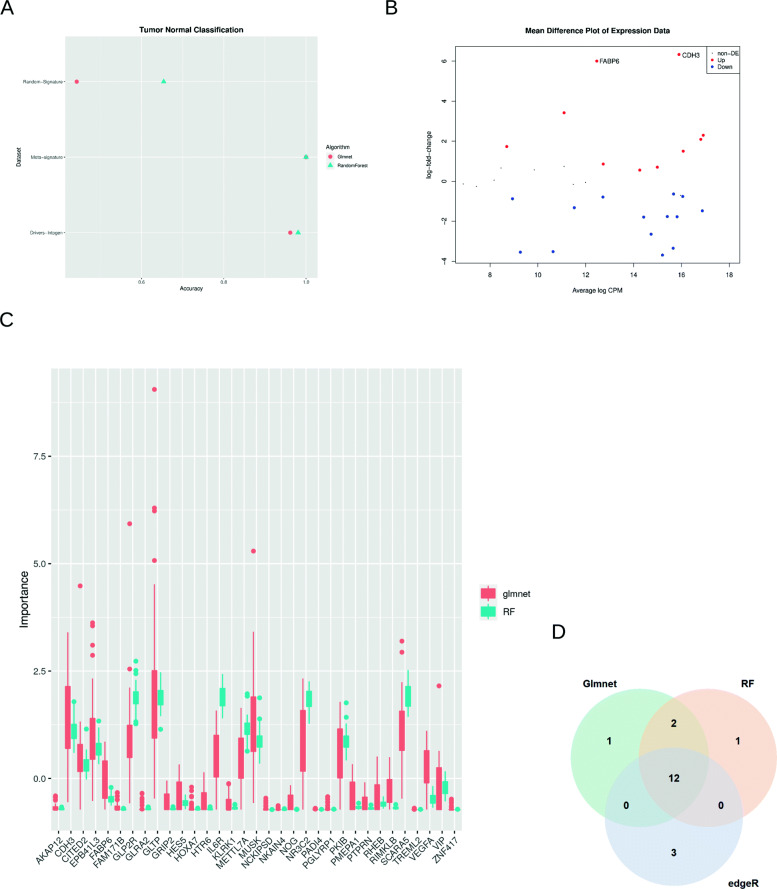
Results of analyisis prediction from tumor and helath tissues. **a**) A comparative ML task was carried out with three different signatures (Random signature, Meta signature and Drivers Intogen) to predict between tumor and helth tissues. TCGA expression values of these three signatures were the input in training phase for two ML algorithms (Random Forest and glmnet). The accuracy of the models for each signature is shown. **b**) Mean difference plot after differencial gene expressión is shown. Up and Down expression genes are highlighted in red and blue respectively. FABP6 and CDH3 were the genes with major gene expression differences. **c**) Comparative variable importance for metasignature in Random Forest and glment algorithms. Values were scaled for comparative analysis. **d**) Pie chart with intersections of same genes obtained by two ML approaches and differential gene expression. The three approaches obtained very similar conclusions

Unlike the previous experiments, we observed in Fig. 3a a greater general precision in this problem. The dataset formed by the genetic drivers presented an almost perfect performance in both algorithms. As for the random signature, its performance dropped considerably. It was also unstable and irregular between the algorithms used and presented a randomness of the results. On the other hand, the meta-signature studied in this work presented a perfect prediction of the samples, surpassing in performance to the signature presented by the genetic drivers. Again, Friedman’s statistical test was performed to observe if there were significant differences between the models. The *p*-value of the test was significant, with a value of 2.2*e*^−16^. Because the test was significant, a multiple comparison test was performed to see which models had a significant difference. The PostHoc Friedman Conover Test variant was used. Assuming a significance level of 0.01, it was determined that the datasets conformed by the meta-signature and the genetic drivers were significantly better than the dataset formed by the random signature. As for the comparison between the first two, there were no significant differences in performance.

We can infer, therefore, that the meta-signature obtained is useful when differentiating patients in a very early stage of the tumour. It is interesting to know at this point of the analysis which of the genes presented a greater weight within the model and a greater discriminatory capacity in the classification between healthy and tumour tissue.

For a further study of the models that were trained with the meta-signature, the importance of the variables in the Random Forest and Glmnet models were extracted. The importance of the variables (standardized to have a mean of zero and a standard deviation of one) within the Glmnet and Random Forest models is shown in Fig. 3c. In addition, Table 2 shows, in descending order, the top 15 most important genes obtained in both algorithms. A differential analysis of gene expression using the package edgeR [29] was also performed on the datasets that presented the variables of the meta-signature. The results obtained in this analysis were compared with those obtained in the ML models (see Fig. 3c and Table 2). In addition, in Fig. 3b, a graph of differential expression obtained by means of the classical approximation is observed. The figure shows how this approximation detected the FABP6 and CDH3 genes as the most significant according to the log fold change. This conventional approach models the data under a negative binomial distribution, calculates the overdispersion coefficient, and performs the exact Fischer Test to obtain the most significant variables. Figure 3d shows a Venn diagram with the coincidences of the three approximations, and it can be seen that the three approximations reach very similar conclusions.

**Table 2:** Top 15 Variable Importance obtained through Glmnet and Random Forest algorithm. In addition, we have compared these results with a classical analysis aproach for differential expression analysis with edgeR package

**Glmnet**	**Random Forest**	**edgeR**
GLTP	GLP2R	CDH3
CDH3	GLTP	GLP2R
MUSK	IL6R	VEGFA
SCARA5	SCARA5	MUSK
NR3C2	NR3C2	PKIB
GLP2R	CDH3	SCARA5
EPB41L3	METTL7A	PMEPA1
PKIB	MUSK	FABP6
IL6R	PKIB	RHEB
METTL7A	EPB41L3	IL6R
CITED2	CITED2	NR3C2
VEGFA	VIP	VIP
FABP6	FABP6	EPB41L3
VIP	VEGFA	GRIP2
RIMKLB	HES5	METTL7A

The results obtained in the analysis of the importance of the variables indicate that the two algorithms and the classical approximation reached almost the same conclusions, and gave importance to the same variables (genes), although there is a small degree of variability. Specifically, among the top 15 variables (genes) of the three approximations, there was coincidence in 80% of them. All of them agree that the genes CDH3, MUSK, SCARA5, NR3C2, GLP2R, EPB41L3, PKIB, IL6R, METTL7A, VEGFA, FABP6 and VIP have great importance when differentiating between healthy samples and tumour samples.

By defining this meta-signature as a predictor in the diagnosis of the disease, and after the results obtained in the different approximations, it is theoretically possible that there exists in this meta-signature, a gene that may have an important role in the development of the tumour and may be a new target for future treatments. For this reason, a molecular affinity study was carried out between the different protein products of these genes and anti-cancer drugs that have been previously approved. In this way, a more in-depth study can be carried out on the results obtained and new specific therapeutic targets for colon cancer can be proposed.

### Molecular docking - drug repurposing

At this point in the work, we found a meta-signature of genes that was able to classify with great precision, healthy and colon cancer tissues. Another important aspect was the importance of the variables in the different models. The three approaches (Random Forest, Glmnet and edgeR) showed great coincidence in the most significant variables, indicating that these genes could have an important role in the disease. In this context, we consider it necessary to conduct an experiment in silico to observe the interactions between the protein products of these genes and various anti-cancer drugs previously approved by the FDA.

**Fig. 4:**
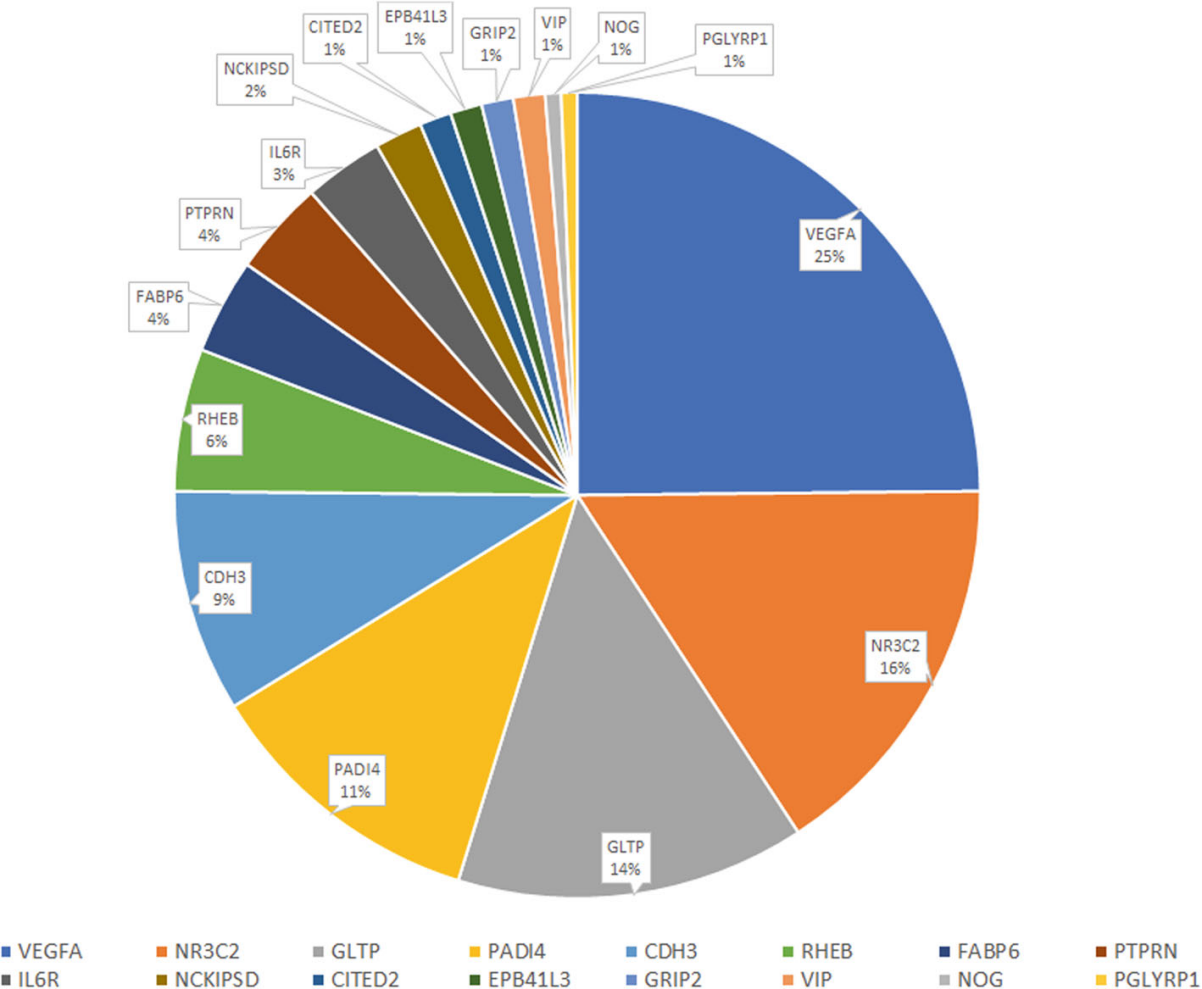
Percentage of 3D PDB structures for each gene obtained

The 34 genes of the signature studied were converted into their different 3D structures, annotated in the PDB repository [30] (only those structures with a validated 3D annotation were chosen). Of these 34 genes, only 16 were 3D annotated: PADI4, VIP, GRIP2, NCKIPSD, PGLYRP1, FABP6, CDH3, VEGFA, NOG, EPB41L3, IL6R, CITED2, NR3C2, RHEB, PTPRN and GLTP. These genes represent 60% of those indicated in Table 2. These 16 genes resulted in a total of 155 PDB structures. Figure 4 shows a diagram representing the number of PDB structures obtained for each gene.

A list of anti-cancer drugs that had been approved by the FDA was selected. In the Supplementary information file S1, a complete list of the name of the 81 drugs corresponding to the compounds downloaded from the ChEMBL repository, which have been used for the Molecular Docking experiment, are shown. All of them are marketed for use in different cancer treatments.

After the execution of the Molecular Docking experiment of confronting the 155 PDB structures with the 81 drugs, the results were obtained for each PDB structure-drug binomial, indicating the value of the interaction in $\frac {kcal}{mol}$. As a result of the study, the 50 strongest interactions (see Supplementary information file S2) were evaluated and only 4 different genes were identified among them, shown in Table 3. For this experiment, a significant interaction was considered for values that were lower than $-7 \frac {kcal}{mol}$.

**Table 3:** Top interactions of the 4 genes that have appeared among the 50 best interactions

**Gene**	**Protein**	**Drug**	**AE (kcal/mol)**
GLTP	3S0I	Nilotinib	-13.7
PTPRN	3NP5	Venetoclax	-12.3
VEGFA	4GLS	Venetoclax	-12.2
FABP6	2MM3	Abemaciclib	-12.1

It is important to point out that among the 50 strongest interactions (see Supplementary information file S2), 92% involved some structure of the GLTP gene. In position 40 and 44 of the ranking, we found PDB structures of the PTPRN gene, in position 48, the PDB structure of the VEGFA gene and in position 49, the PDB structure of the FABP6 gene. Although there is a predominance of PDB structures of the GLTP gene, there is little difference in the force of interaction, showing a decrease of only $-1.6 \frac {kcal}{mol}$ between the strongest interaction (3SOI-Nilotinib) and the one in position 49 (2MM3-Abemaciclib).

A review study was made for each of these four genes to see which might be possible therapeutic targets.

### Study of each of the genes

**Fig. 5:**
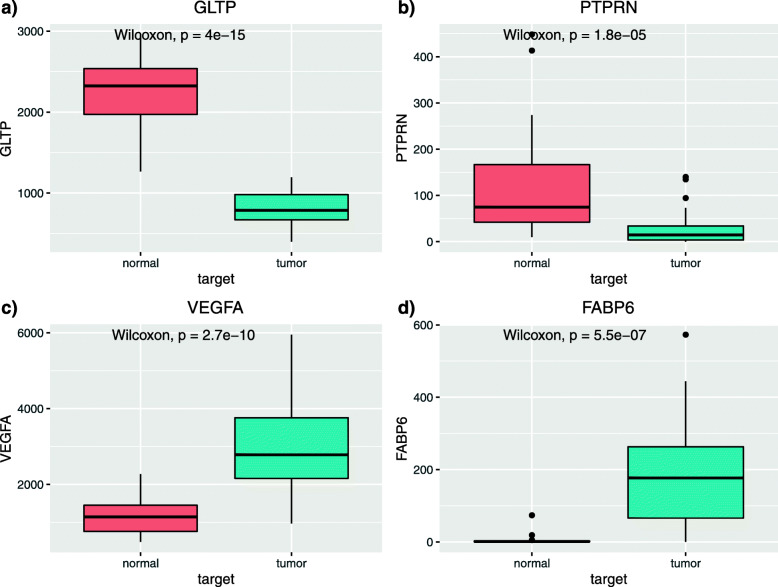
Box diagram of the expression of the four genes between healthy and diseased tissues of the COAD cohort of the TCGA

A comparative study of the four genes was carried out to analyse if any of them could behave as a possible therapeutic target. In Fig. 5 the expression between tumour tissue and adjacent healthy tissue of each of the four genes from the COAD cohort of the TCGA is seen.

As can be seen, the genes GLTP and PTPRN present an underexpression in tumour tissue, so attacking it through inhibitor drugs will not produce a positive consequence when slowing tumour development. On the other hand, VEGFA and FABP6 genes are overexpressed in tumour tissue, which makes them possible candidates for inhibitory therapy. This is an important step because in addition to observing whether the gene is over- or under-expressed in tumour tissue, it is crucial to know what its status is in normal tissue. As shown in Fig. 5, VEGFA has significant expression in normal tissue. Whereas the FABP6 gene showed no expression in normal tissue, which is beneficial if our objective was to propose it as a possible biomarker and therapeutic target. Therefore, the biological function of this gene has been deepened.

Docking studies show that the drugs *venetoclax* and *abemaciclib* (previously known as LY2835219) have a significant interaction with the VEGFA and FABP6 genes, respectively. As for the drug *venetoclax*, itwas approved in 2016 as therapy for patients with Chronic Lymphocytic Leukemia (CLL). The mechanism of action of this drug focuses on inhibition of the apoptosis regulator Bcl-2, which is a ’single protein’ [31]. Moreover, textitabemaciclib was approved in 2017 for breast cancer patients. Like the previous drug, this is an inhibitor against cyclin-dependent kinase 6, which is also a ’single protein’ [32].

In this way we ruled out genes underexpressed in tumour due to the type of drugs we tested. As for the VEGFA and FABP6 genes, the first of them (Vascular Endothelial Growth Factor A - VEGFA) is a specific growth factor for vascular endothelial cells, capable of inducing angiogenesis in vivo [33]. This gene is the central axis in tumour angiogenesis, and there are already different experimental therapies tested against this gene [34, 35]. In addition, different studies are working to predict the different peptides, in silico form, that act against this target [36–39].

The FABP6 gene produces Ileal lipid-binding protein (ILBP) which is a member of a family of fatty acid binding proteins, retinoic acids, and intracellular bile acids [40]. In relation to cancer, the FABP family has been reported to play a role in the development and pathogenesis of cancer [41], and as a possible therapeutic target in clear renal cell carcinoma [42]. Specifically, the FABP6 gene has been suggested as a potential drug discovery target [24, 43], although to date no therapy directed against this gene and/or protein product has been approved.

Our findings are in line with the conclusions shown in the work of Ohmachi et al. [23] published in 2006 by a high impact journal such as Clinical Cancer Research. In this work, they observed that the expression of FABP6 was higher in primary colorectal cancers and adenomas than in normal epithelium, thus suggesting that FABP6 plays an important role in early carcinogenesis. The results of our research are linked to this conclusion, firstly by observing how our signature, in which FABP6 was present, was able to predict more accurately, even more than previously identified genetic drivers, between healthy and tumoural tissue. In addition, analysis of the importance of variables in ML models and differential expression analysis showed that FABP6 was at the top of both lists (see 3 b and c).

Omachi et al. [23] also focused their research on the FABP6 gene because of the large difference in gene expression between healthy tissue and tumor tissue. In addition, the results of [23] were based on a Chinese population, while ours are from the USA. It can be inferred that the function of this gene could be cross-sectional in different world populations. These differences are explained by the high concentration of secondary bile acids present in patients with colonic adenoma.

Reinforcing our hypothesis that FABP6 may be an interesting biomarker for colon cancer, in the same work Ohmachi et al.[23] found that tumours expressing higher levels of FABP6 were smaller, supporting that theory that FABP6 could be a biomarker for the early stage of carcinogenesis.

In addition to being an early stage marker in COAD, we believe that FABP6 may also behave as a therapeutic target because: 1) it is known that each of the nine types of FABP proteins shows tissue specificity, with FABP6 being the ileum, thus generating specificity in future treatment; and 2) the expression of FABP6 in tumour tissue is due to an increase in secondary bile acids, and it is known that the action of these bile acids triggers cellular apoptosis [44]. Therefore, avoiding the metabolisation of these acids would cause apoptosis in cells of the cancerous tissue, abruptly stopping their growth.

Therefore, the data we found in the literature led us to design a drug repurposing experiment to find an already approved drug that could specifically bind to this possible therapeutic target.

### Deepening in Abemaciclib and FABP6 interaction

The Molecular Docking results presented in this work show a significant specificity of all protein PDB structures of the FABP6 gene with the drug *Abemaciclib*. Our experiment took into account a total of six PDB structures (2MM3, 1O1U, 1O1V, 5L8I, 5L8N, 5L8O). In Table 4, the interaction force of the *Abemaciclib* with all the PDB structures annotated in the FABP6 gene is shown. As shown in the Table 4, all interaction forces have a value of less than -7 *kcal*/*mol*, and all are considered significant.

**Table 4:** Interaction force of Abemaciclib with all PDB structures of the FABP6 gene

**Gene**	**PDB structure**	**Drug**	**AE (kcal/mol)**
FABP6	2MM3	Abemaciclib	-12.1
FABP6	1O1U	Abemaciclib	-8.0
FABP6	1O1V	Abemaciclib	-10
FABP6	5L8I	Abemaciclib	-9.0
FABP6	5L8N	Abemaciclib	-9.5
FABP6	5L8O	Abemaciclib	-10

In order to understand the docking details, a new open science Web tool was introduced as COAD-DRD: Colon Adenocarcinoma Drug Repurposing with Docking, available at https://muntisa.github.io/COAD-DRD/. There are six different sections: Abemaciclib-FABP6 - dedicated to the interactions of Abemaciclib with the six PDB structures, Selected - with the most interesting interactions between drugs and genes in COAD, Top50 - with statistical plots about the docking signature of the best 50 drugs in COAD, Full by Genes - with statistical box plot for the interactions of each gene with all the drugs (without any cutoff for the AE), Full by Drugs - with graphics that show the drug signature on all the genes in COAD, and Full DB - a pivot table with graphics that give the possibility to represent all docking results by any criteria.

COAD-DRD sections provide interactive graphics, interactive 3D structures for the complexes that provide direct visualisation with the binding poses, interactive tables with specific datasets for each section (with filtering, searching), interactive pivot tables with a high degree of flexibility to visualize the entire dataset for this study, and direct visualisation of important docking information (docking outputs, search box configuration, pdbqt files, pictures of interactions, contact atoms, or hydrogen bonds, etc.). The source code and all the other files, including the script to generate the dynamic elements, are available as an open GitHub repository at https://github.com/muntisa/muntisa.github.io/tree/master/COAD-DRD.

Based on our findings, the FABP6 gene and, specifically, its protein products, are proposed as therapeutic targets for the development of colon cancer. In addition, owing to the drug repurposing experiment, we present the drug *abemaciclib* as a possible drug that may interact specifically against the protein products of this gene.

**Fig. 6:**
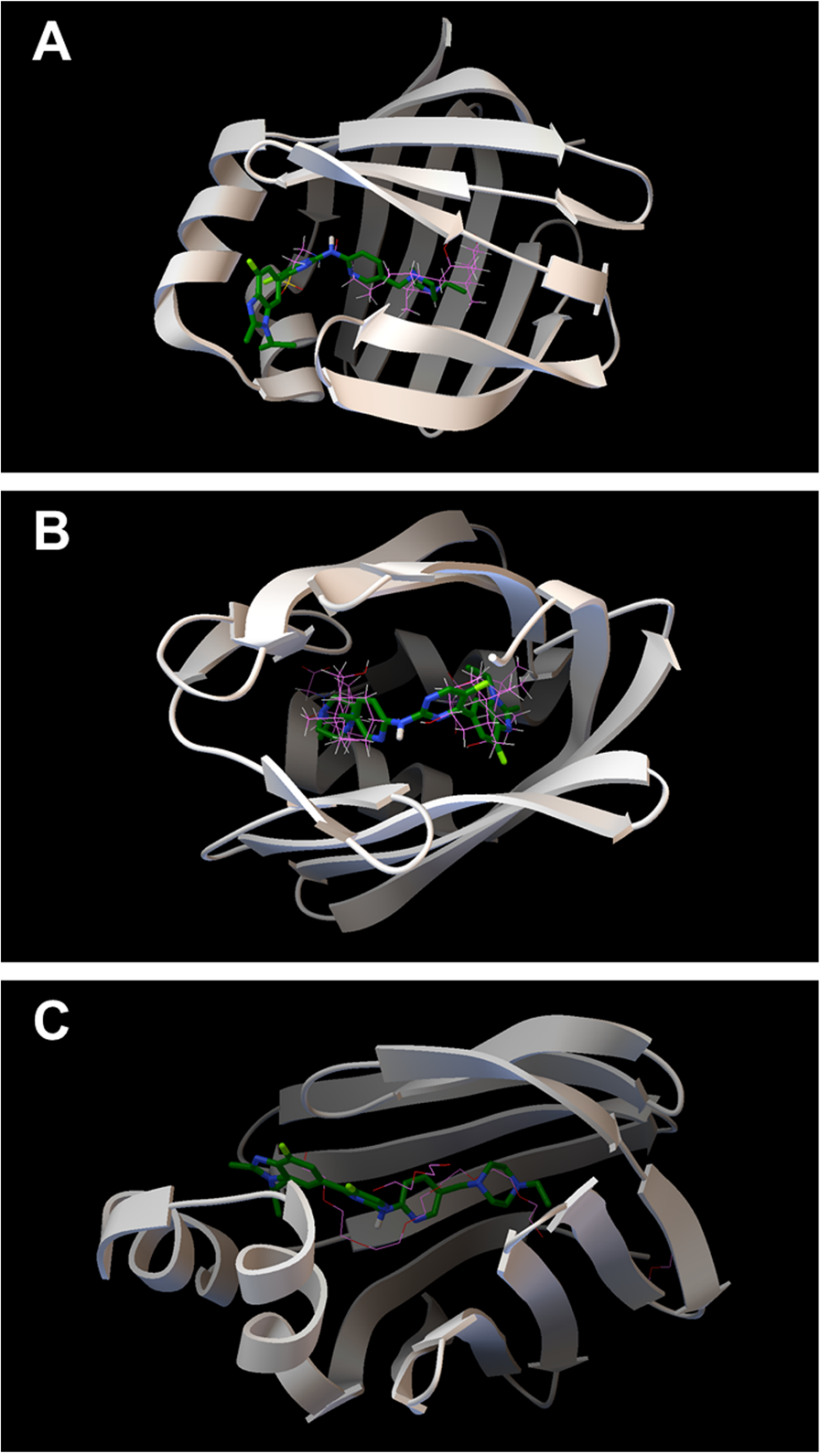
Three FABP structures (white ribbon) with the natural ligands (violet lines) and Abemaciclib (blue-green sticks and balls): 1O1V **a**, 2MM3 **b**, and 5L8N **c**

Blind molecular docking means that the search of the best Abemaciclib interaction uses the entire surface of the FABPs without defining an active site of the natural ligands (lipids). This could generate docking results where Abemaciclib could interact out of the active site without implications in the FABP activity. Therefore, Fig. 6 presents the three FABP structures with the natural ligands and the best interaction of Abemaciclib with the FABPs in order to check the location of these interactions. FABPs are represented using ribbons (white), the natural ligand using lines (violet) and Abemaciclib using sticks and balls (blue-green).

Figure 6 presents FABP with PDB ID 1O1V: human ileal lipid-binding protein (ILBP) in complex with cholyltaurine (ligand). This protein has a single ligand active site defined by 10 amino acids: TYR14, MET18, ILE23, VAL27, TRP49, TYR53, ASN61, MET74, LEU90, ARG121. 1O1V amino acid – Abemaciclib atom interactions are SER54:HG, MET59:CE, ILE23:CD1, TYR53:CE2, VAL27:CG1, ILE23:CG2, LYS77:CD, GLN51:HE21, VAL27:CG2, MET74:CG, SER24:H, TYR53:CD2, TYR14:OH, VAL27:CB. Thus, Abemaciclib interacts with TYR14, ILE23, SER24, VAL27, GLN51, TYR53, SER54, MET59, MET74, LYS77. From these amino acids, five of them are defining the active site of 1O1V: TYR14, ILE23, VAL27, TYR53, and MET74. In addition, the visualization of Fig. 6a demonstrated that Abemaciclib occupy the FABP active site, with consequences in the lipid transport activity.

Figure 6b presents FABP with PDB ID 2MM3: human ileal bile acid-binding protein with glycocholate and glycochenodeoxycholate (two ligands). This protein has two active sites for the two ligands. AC1 site for glycocholate ligand (CHO202) is defined by 22 amino acids: PHE2, PHE6, MET8, MET18, ALA31, ILE36, THR38, VAL40, PHE47, TRP49, GLN51, MET74, LEU90, SER101, GLU102, LEU108, VAL109, GLU110, TYR119, ARG121, and SER123. AC2 site for glycochenodeoxycholate ligand (GCH201) is defined by 17 amino acids: LEU21, ILE23, TRP49, ASN61, PHE63, GLN72, THR73, MET74, GLY75, LYS77, PHE79, VAL83, LEU90, VAL92, TYR97, and GLN99. 2MM3 amino acid – Abemaciclib atom interactions are PHE47:CZ, GLN99:HE21, MET8:CB, PHE47:CE1, GLN99:NE2, PHE2:CB, PHE47:CE2, THR38:CB, GLN51:HE21, PHE2:CD2, PHE79:CZ, LEU90:CB, GLN51:NE2, GLN99:CD, TYR97:CE2, ARG121:CD, VAL109:N, SER101:CB, TRP49:CZ3, VAL109:C, MET8:CG, SER101:HG, THR38:CG2, PHE2:C. Thus, Abemaciclib interacts with PHE2, MET8, THR38, PHE47, TRP49, GLN51, PHE79, LEU90, TYR97, GLN99, SER101, VAL109, and ARG121. All these amino acids are defining both active sites for both natural ligands in 2MM3: PHE2, MET8, THR38, PHE47, GLN51, SER101, VAL109, ARG121 from AC1 active site and TRP49, PHE79, LEU90, TYR97, GLN99 from AC2 active site. Figure 6b shows that Abemaciclib occupy both FABP active site in the same time. This interaction should disturb the both lipid transport activity.

Figure 6c presents FABP with PDB ID 5L8N: human FABP6 protein with fragment 1 + 3,6,9,12,15,18-hexaoxaicosane-1,20-diol (P33); 5,6-dimethyl-1 H-benzimidazol-2-amine; di(hydroxyethyl)ether (PEG). This protein has several active sites and only the one for P33 and PEG will be compared with the Abemaciclib interaction preference: AC5 site is defined by 11 amino acids - PHE18, TRP50, ILE72, THR74, GLY76, LEU91, TYR98, GLN100, THR101, SER102, ARG122. 5L8N amino acid – Abemaciclib atom interactions are ALA32:CB, PHE64:CZ, MET19:CE, LEU91:CB, VAL28:CG1, PHE64:CE2, GLN100:NE2, TRP50:CE2, TRP50:CG, GLY76:CA, ILE72:CD1, TRP50:CD2, and MET75:CB. Thus, Abemaciclib interacts with MET19, VAL28, ALA32, TRP50, PHE64, ILE72, MET75, GLY76, LEU91, and GLN100. Five of these amino acids are defining AC5 active sites in 5L8N: TRP50, ILE72, GLY76, LEU91, and GLN100. Fig. 6c shows that Abemaciclib occupy the FABP active site where normally interacts both ligands: P33 and PEG. This interaction should modify the ability of FABP to transport lipids.

In conclusion, Abemaciclib prefers interactions inside the active site of FABPs using more nonpolar/aliphatic/hydrophobic amino acids (GLY, ALA, VAL, LEU, ILE, MET, TRP, PHE) than hydrophilic uncharged amino acids (SER, THR, TYR, GLN) or charged/basic amino acids (LYS, ARG). This is explained by the FABP preference for aliphatic interactions to link natural lipids for transport.

The results will then be discussed, focusing mainly on the interaction of FABP6 and *abemaciclib* protein products. Proposing in this way, a new potential candidate to be validated experimentally.

## Discussion

The results obtained in this work report the FABP6 gene as a possible therapeutic target and the drug *abemaciclib* as a possible drug directed towards the gene products of this gene.

As mentioned above, this gene functions as a receptor for lipids and bile acids. It is curious to observe how its genetic expression is almost totally specific to the small intestine - terminal ileum, with average values around 500 RPKM. In other tissues, as can be seen in the data from the Genome Browser [45], its expression is practically null. Therefore, the data shown in Fig. 5 indicate an abnormal function of that gene in carcinogenic tissue.

**Fig. 7:**
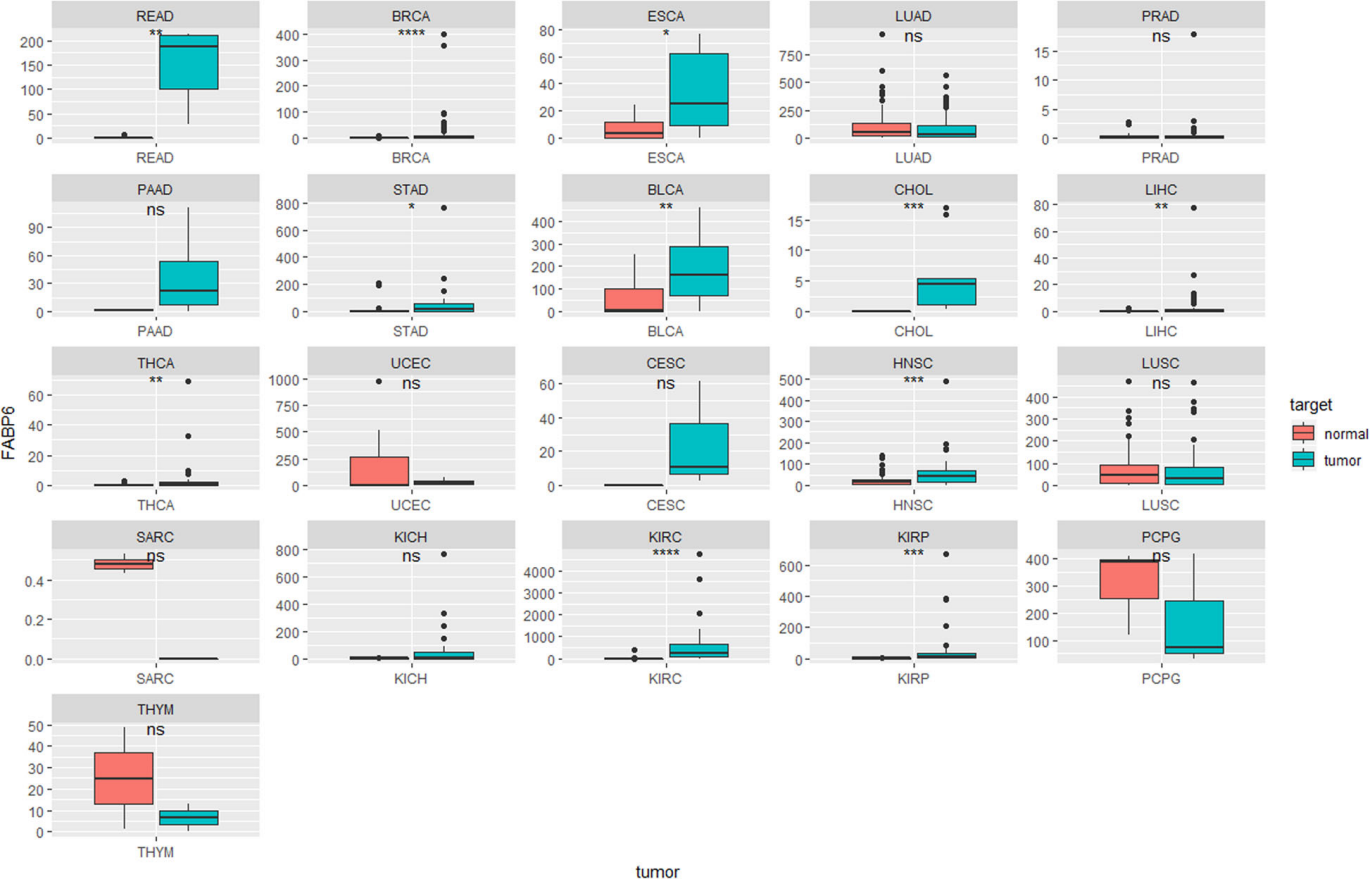
Box plot panel with the comparision between tumour and control samples through 21 tumour s types from TCGA

It is interesting to note the expression behaviour of this gene in different tumour. With the available TCGA data, the expression of the FABP6 gene in healthy and tumour tissue has been compared (see Fig. 7).

The results show a significant difference in certain types of tumour. Firstly, we observed how the expression is practically null in all the healthy tissues of each one of the different patients, being indifferent to the type of cancer. However, the PCPG cohort shows some contradictory results to what was previously proposed, which could harbour new functions and roles of the FABP6 gene. On the other hand, although there is a significant difference in certain tumours (as may be the case of the breast adenocarcinoma (BRCA), stomach adenocarcinoma (STAD) or cholangiocarcinoma (CHOL) cohort, for example), this difference is mainly due to the outliers, as shown in the different box diagrams. This does not occur in the READ cohort (rectum adenocarcinoma), which presents high levels of FABP6 in tumour tissues throughout the sample. This fact coincides with the results shown in this work, supporting the idea of specificity of FABP6 expression in colorectal tissue.

In this context, we can conclude that FABP6 is a specific biomarker for COAD and READ, so the action of an inhibitory mechanism could lead to positive results in slowing down the growth of the tumour. Furthermore, as mentioned above, FABP6 is an early diagnostic biomarker, which would greatly assist the various possible treatments of this type of cancer.

Regarding its function, this gene intervenes mainly in the signalling peroxisome proliferator-activated receptor (PPAR) pathway. The FABP family activates the PPAR signalling pathway, which acts as transcription factors, regulating the expression of different genes related to lipid metabolism, adipocytic differentiation, adaptive thermogenesis, cell survival, gluconeogenesis and ubiquitination [46]. These functions may be related to the development and differentiation of cancer cells. In addition, previous studies have already linked this pathway to cancer, and specifically to colon cancer [47–50].

**Fig. 8:**
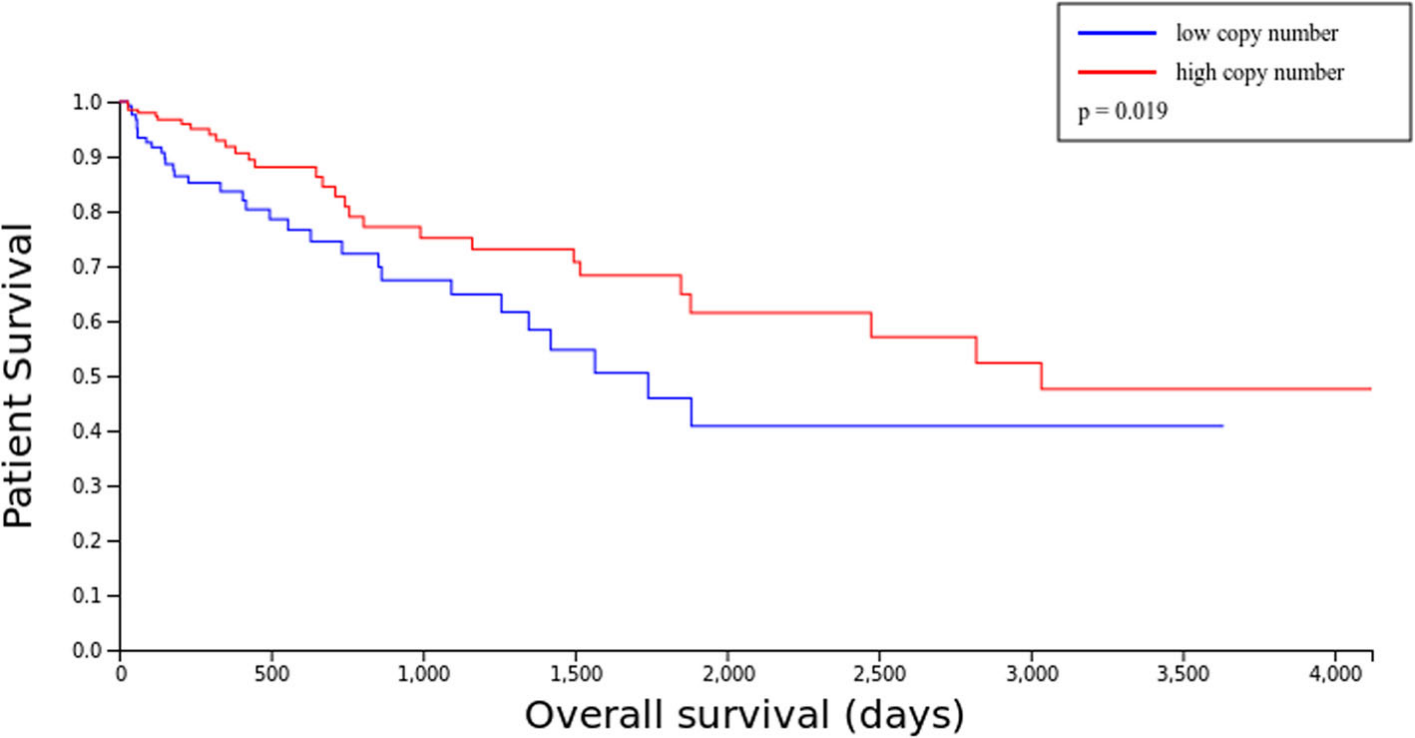
Survival curve according to the number of copies of the FABP6 gene. Extracted from [52]

Comparisons with other studies and databases, show a significant decrease in the survival of patients with a high copy number of the FABP6 gene, as seen in Fig. 8, obtained from the web and article [51, 52]. From this survival curve and the function of the gene, it can be inferred that when there is a very abrupt change in the coding of the FABP6 gene, there can be serious problems in the survival of the patient. Due to its function of regulating fatty acids and bile acids, and after development of COAD, the patient will present greater deregulation in gastrointestinal homeostasis, which would justify its worst prognosis. At this point, it is interesting to point out that the aberration or inhibition of this gene in tumour cells alone could theoretically provide an advantage when considering this gene as a possible therapeutic target. Due to the need for tumour cells to provide continuous energy, the metabolic pathways related to fatty acids must be expressed in a significant way. Deregulation of these cellular pathways could provide detection of the growth and development of the tumour. This annotation could justify the results, previously commented on in the article [42].

On the other hand, the drug selected as the ligand for this gene, *Abemaciclib*, has reasonable characteristics to be used as a drug against this type of cancer. It is a small molecule specific inhibitor of cyclin-dependent kinase 4/6, so its effect lies in the detection of cell division by acting on the regulation of G1 phase of the cell cycle. It was approved for use in breast cancer patients in 2017. Although there are no results from clinical trials for this type of cancer and this drug, large pharmaceutical companies are already testing it in combination with other drugs, such as *Ramucirumab* for patients with advanced cancer, colon cancer, and mantle cell lymphoma [53], which also supports our hypothesis.

Finally, and after the evidence gathered, both in our own experiments and in previous work, the FABP6 gene and the drug *Abemaciclib* are proposed as a possible targets and treatment, respectively, in colon cancer. The effect of the drug on other types of cancer, as well as the results obtained in this work, support the hypothesis put forward by the present researchers that this drug will join CDK4/6 and FABP6 protein products (specifically in carcinogenic tissue due to its low expression in different tissues), inhibiting both functions and therefore significantly reducing the development of cancer. Although this hypothesis must be validated experimentally, there is sufficient theoretical evidence to think of the gene and the drug as potential anti-cancer therapies.

## Conclusions

The results in silico of this work show how the drug *Abemaciclib*, previously approved for treatment in breast cancer could be used, a priori, in the treatment of colon cancer. In breast cancer, Abemeciclib inhibits CDK4/6, interrupting the cell cycle and the development of the tumour. In this work, we report that this drug could also be used for the treatment of colon cancer, after subsequent experimental validations, due to its strong interaction with all the protein PDB structures of the FABP6 gene. A thorough comparative study was carried out, to observe the evidences that existed after the inhibition of this protein product. All of the evidence indicates that inhibition of the expression of the FABP6 gene, specifically in tumour cells, would reduce the development and growth of the tumour.

This work demonstrates that in silico techniques, such as Machine Learning and Molecular Docking techniques, create added value to the data reported by other initiatives. Owing to the reuse of free access data, it is possible to use computational methods to validate, test, and prove a hypotheses, and thereby considerably reduce research costs.

Finally, in order to obtain new alternatives in the treatment of cancer, the presented hypothesis need to be experimentally validated in the laboratory.

## Methods

### Datasets

RNASeq2 data from the COAD cohort was downloaded from the TCGA repository [3] using the TCGA2STAT package [54]. Patients were filtered according to the type of problem being studied. For classification according to the metastatic status in the lymph nodes, a total of 283 patients were obtained, classifying 166 in stage *N*_0_ and 117 between stages *N*_1_ and *N*_3_.

For the disease stage classification problem, 154 patients were classified between the stages *S*_1_ and *S*_2_, while 120 were classified between the stages *S*_3_ and *S*_4_.

Finally, for the problem of classification between healthy and tumour tissues, 26 patients were included in the analysis. These patients presented RNASeq data of their tumour tissue and adjacent normal tissue. For a better understanding of this cohort, some of the clinical data of these patients can be seen Supplementary information file S3.

### Differential expression analysis

A differential expression analysis was performed using the edgeR package. This package assumes that the number of readings in each sample (j) assigned to a gene (i) is modeled through a binomial negative distribution with two parameters, the mean *μ*_*i*_, j and the overdispersion parameter *Θ*_*ij*_.

$$Y_{ij} ~ BN\left(u_{ij}, \Theta_{ij}\right)$$

*Y*_*ij*_ corresponds to the non-negative whole number of readings in each sample (j) assigned to a gene (i). The values of the mean and the overdispersion, in practice, are not known so we must estimate them from the data. Finally, using the exact test for the negative binomial distribution, differentially expressed genes are estimated.

### Machine learning

The following algorithms were implemented: random forest (RF) and generalized linear model (glmnet). A nested cross validation was used for training the models. In other words, there were two validation phases. Firstly, a holdout was used for the selection of the best hyperparameters (2/3 for training and 1/3 for testing) and secondly, a Leave One Out was used for the validation of the model.

### Molecular docking

The strength of the interactions were quantified by the affinity energy (AE, kcal/mol) of ligands for protein targets using the open software AutoDock Vina [55]. The entire processing was done into the BioCAI cluster from the University of A Coruna (Spain). The docking flow had several steps that included the ligand and protein processing, conversion and geometry optimisation before the docking calculations.

Thus, the ligands are presented as a list of commercial drug names. Using PubChem APIs, the compounds for all drugs have been downloaded as SDF 2D. The ligand molecules were converted into PDB by optimising the 3D structure using babel software [56]. The protein targets were only filtered for the first PDB model, the non-protein part was eliminated (water molecules, other ligands, etc.). The PDB of ligands and proteins were converted into PDBQT format using AutoDockTools scripts (prepare_ligand4.py and prepare_receptor4.py) [57]. The protein target was considered rigid in all docking calculations and the interaction searching was considering the entire surface of the targets. The docking flow is based on python and bash scripts, including the reading of the final results. The cut-off for stable interactions is considered AE $< -7.0 \frac {kcal}{mol}$ [58]. The results are based on the first docking conformer of the ligands with reference root-mean-square deviation of atomic positions (RMSD) of 0 [59]. We are presenting the top 50 interactions (the most negative AE values). We used 155 protein targets and 151 compounds (24.273 dockings/AE values). The list of interactions and the docking figures are presented for one of the best interaction such as nilotinib – compound 644241 (ligand) with 3s0i (protein target).

In order to understand all the details, a new open Web tool was introduced as COAD-DRD: Colon Adenocarcinoma Drug Repurposing with Docking (https://muntisa.github.io/COAD-DRD/). The tool includes several sections about the best proposed drug for COAD, the top 50 interactions, our selection of interaction and the entire dataset of docking results. All the files and the source of the tool is available as an open GitHub repository at https://github.com/muntisa/muntisa.github.io/tree/master/COAD-DRD. The sections of the web included interactive tables, plots, pivot table and 3D structures widgets (generated with python jupyter notebooks based on HTML, plotly - https://plotly.com, ipywidgets - https://ipywidgets.readthedocs.io/en/latest/, nglview - https://github.com/arose/nglview (DOI:10.5281/zenodo.3700850), pivottablejs - https://pivottable.js.org and datatables - https://datatables.net). Thus, it is possible to zoom into the 3D complex structures between drug binding poses and targets, search for specific results, find details into plots, understand the drug signature on all COAD genes, check the contact atoms and hydrogen bonds of the interactions, and download all docking files.

### Analysis pipeline

In this section we will describe the pipeline followed to obtain the candidates for genes presented in this work. Next, each of the stages carried out in this work will be described step by step.

#### State of the art review

The objective of this work consisted of the search and validation of signatures and therapeutic targets for colorectal cancer already reported in the literature.

To this end, a review of published papers that have used TCGA data for the execution of Machine Learning algorithms was carried out. Of all the works found, only those studies in which the dependent variable was related to the prognosis of the disease were chosen. Finally, three papers were identified. Each of these studies reported a signature of genes related to the prognosis of colon cancer patients.

#### Generation of the meta-signature

Secondly, a gene signature was built by merging the three previously identified signatures. A total of 34 genes were obtained. The signature was checked for previously defined drivers for colon cancer. For this purpose, the drivers defined in colon cancer were downloaded from the Intogen database, and no coincidence was found between the two lists.

If we assume that the expression of these genes influences the prognosis of individuals, it is interesting, firstly, to know if this signature accurately predicts the prognosis of patients in a cohort such as TCGA and secondly, if any of these genes could be a future protein target, which could be attacked with drugs already approved in the industry.

The signature was then validated for two different types of problems. Firstly for the classification of the stage of cancer, and secondly, for the classification of patients between healthy and sick. This experiment was followed by a study of the importance of the variables within the best models.

#### Search for new therapeutic targets

The next focus in this work was the detection of possible new therapeutic targets using drug repurposing. This experiment presents two well-differentiated parts: the obtaining of targets (proteins) and the obtaining of ligands (drugs).

In order to obtain the targets, the signature of genes (HGNC nomenclature) and all of its possible protein PDB structures were transformed. The transformation was carried out through the biomaRt package. In this step, part of the genes were lost because there is no annotation in PDB for all the protein products of all the genes. In the end, we were left with 16 genes that do have PDB annotation. In the Table 5, the list of genes used for the Molecular Docking experiment is shown. A total of 155 PDB structures derived from these genes were analysed.

**Table 5:** List of genes, with PDB annotation, used for the Molecular Docking experiment

PADI4	VIP	GRIP2	NCKIPSD
PGLYRP1	FABP6	CDH3	VEGFA
NOG	EPB41L3	IL6R	CITED2
NR3C2	RHEB	PTPRN	GLTP

To obtain the ligands, anti-cancer drugs that have already been approved for treatment were chosen. The objective of this process was to find a drug, already approved, that has a significant interaction force against a protein target in order to reuse it, in this case, for colon cancer.

The anti-cancer drugs were obtained from the website of the National Cancer Institute [60]. To validate all the names of the drugs, they were downloaded from the repository DRUG REPURPOSING HUB [61]. We made a combination of both lists and only kept those that were already passed the clinical trial and, therefore, are in the market. Finally, after processing, we were left with 81 approved anti-cancer drugs.

## Supplementary information

**Additional file 1** Additional file S1. List of ligands with their corresponding annotation in ChEMBL. It showed a list of ligand used in the docking experiment. A total of 159 ChEMBL coumpounds are listed from 81 anti-cancer drugs downloaded.

**Additional file 2** Additional file S2. Top 50 interactions from docking experiment. In this excel file it can be found the interaction force between ligand (drug) and target (protein). The interaction force is measured by kcal/mol.

**Additional file 3** Additional file S3. Clinical data of patients involved in classification between tumour and health tissue. In this excel file it can be found several clinical variables that correspond to patients involved in tumour and health tissue classification.

## Data Availability

The datasets analysed during the current study are available in the TCGA repository.

## Abbreviations

AE: affinity energy
AECC: Spanish Cancer Association
AIDS: Acquired Immunodeficiency Syndrome
BLCA: Urothelial Bladder Carcinoma
BRCA: Breast Invasive Carcinoma
CCRCC: Clear Renal Cell Carcinoma
CDK4/6: Cyclin-dependent kinase 4/6
CESC: Cervical Squamous Cell Carcinoma and Endocervical Adenocarcinoma
CHOL: Cholangiocarcinoma
CLL: Chronic Lymphocytic Leukemia
COAD: Colon Adenocarcinoma
ESCA: Esophageal Carcinoma
FDA: Food and Drug Administration
GLMNET: Generalized Linear Model
HIV: Human Immunodeficiency Virus
HNGRI: The National Human Genome Research Institute
HNSC: Head-Neck Squamous Cell Carcinoma
ILBP: Ileal lipid-binding protein
KICH: Kidney Chromophobe
KIRC: Kidney Renal Clear Cell Carcinoma
KIRP: Cervical Kidney renal papillary cell carcinoma
LIHC: Liver Hepatocellular Carcinoma
LUAD: Lung Adenocarcinoma
LUSC: Lung Squamous Cell Carcinoma
MD: Molecular Docking
ML: Machine Learning
NCI: National Cancer Institute
PAAD: Pancreatic adenocarcinoma
PCPG: Pheochromocytoma and Paraganglioma
PDB: Protein Data Bank
PPAR: Peroxisome Proliferator-Activated Receptor
PRAD: Prostate Adenocarcinoma
READ: Rectum Adenocarcinoma
RF: Random Forest
RPKM: Reads Per Kilobase Million
SARC: Sarcoma
STAD: Stomach Adenocarcinoma TCGA: The Cancer Genome Atlas
THCA: Thyroid Cancer
THYM: Thymoma
UCEC: Uterine Corpus Endometrial Carcinoma
